# Temporal Bone Osteomyelitis: The Relationship with Malignant Otitis Externa, the Diagnostic Dilemma, and Changing Trends

**DOI:** 10.1155/2014/591714

**Published:** 2014-05-18

**Authors:** Jia-Cheng Chen, Chien-Fu Yeh, An-Suey Shiao, Tzong-Yang Tu

**Affiliations:** ^1^Department of Otolaryngology, Taipei Veterans General Hospital, No. 201, Section 2, Shipai Road, Beitou District, Taipei City 11217, Taiwan; ^2^Department of Otolaryngology, National Yang Ming University, Taipei, Taiwan; ^3^Department of Otolaryngology, Taipei Veterans General Hospital, Su-Ao and Yuan-Shan Branch, Yilan, Taiwan

## Abstract

Fifty-five patients hospitalized for osteomyelitis of the temporal bone between 1990 and 2011 were divided into two study groups: group 1 was patients collected from 1990 to 2001 and group 2 was composed of patients between 2002 and 2011. Clinical diagnostic criteria and epidemiologic data were analyzed to illustrate the altering features of osteomyelitis of the temporal bone. Group 1 patients were characterized by high prevalence of diabetes and more commonly suffered from otalgia, otitis externa and granulation tissue in the external auditory canal and higher positive culture for *Pseudomonas aeruginosa*. Noticeable changing trends were found between both groups, including declining prevalence of diabetes, fewer patients complaining of pain or presenting with otitis externa, and canal granulation, and increased variety of pathogens in group 2. We should highlight the index of clinical suspicion for osteomyelitis of the temporal bone, even in nondiabetic or immunocompetent patients. Painless otorrhea patients were also at risk of osteomyelitis of the temporal bone, especially patients with previous otologic operation. Increased multiplicity of pathogens amplified the difficulty of diagnosis for osteomyelitis of the temporal bone.

## 1. Introduction

Osteomyelitis of the temporal bone is a rare but life-threatening complication that is mostly secondary to malignant otitis externa (MOE) and was first recognized as a distinct clinical entity by Meltzer and Kelemen in 1959 [1]. Chandler presented the first comprehensive description in a case series from 1968 to 1974 [2]. Most cases (86–90%) have been reported in diabetic patients. There is increased pH in diabetic cerumen, and diabetics have innate defects in chemotaxis and phagocytosis of polymorphonuclear lymphocytes, monocytes, and macrophages, which may contribute to the development of MOE [3, 4]. Since both aging and diabetes mellitus are associated with abnormalities of small blood vessels, it has been postulated that microangiopathy in the ear canal predisposes elderly diabetic patients to MOE [5]. The most commonly causative organism is* Pseudomonas aeruginosa*, although other organisms such as* Proteus mirabilis*,* Aspergillus fumigatus*,* Proteus* sp.,* Klebsiella *sp., and* staphylococci* have been isolated [6]. Typically, the disease begins as an infection of the soft tissues surrounding the external auditory canal, with characteristic symptoms of severe unremitting throbbing otalgia, purulent otorrhea, the sensation of a blocked ear, and hearing loss [7]. Then, spread of infection into the temporal bone occurs through the fissures of Santorini and the tympanomastoid suture, leading to involvement of the stylomastoid and jugular foramina and eventually affecting cranial nerve function. The diagnosis of osteomyelitis of the temporal bone is based on a combination of clinical findings, a laboratory test, radiographic examination, and nuclear imaging. In 1987, Cohen and Friedman suggested a diagnostic scheme to help differentiate between uncomplicated otitis externa and MOE [8]. However, the authors experienced that the varying clinical features and bacteriology had been produced to increase difficulty in diagnosis and treatment for osteomyelitis of the temporal bone in recent years. Yet there were few reports mentioning these changes [9, 10]. In the current study, we investigated the following: first, whether osteomyelitis of the temporal bone always resulted from MOE and if the criteria of Cohen and Friedman could be applied to the diagnosis of osteomyelitis of the temporal bone; second, the changing traits of osteomyelitis of the temporal bone in the past 10 years, including clinical manifestations and bacteriology; and, finally, the appropriate diagnostic strategies for the new faces of osteomyelitis of the temporal bone. In this study, we analyzed the major changes in etiology and epidemiology of this evolving disease in our hospital between two decades and highlighted the need for a high index of suspicion, especially in patients with atypical clinical presentation.

## 2. Materials and Methods

This was a retrospective review of the charts of 55 hospitalized patients with diagnosis of osteomyelitis of the temporal bone who were treated in the Department of Otolaryngology, Taipei Veterans General Hospital, from January 1990 to December 2011. The criteria on which the diagnosis of osteomyelitis of the temporal bone was based included (1) clinical symptoms of persistent otalgia or otorrhea; (2) failure to respond to medical therapy and local treatment; (3) uptake at the temporal bone on bone scan and gallium scan; (4) exclusion of ear tumor by histopathology if granulation tissue was present; and (5) positive findings on computed tomography (CT) or magnetic resonance imaging (MRI). These patients were divided to two groups. Patients who were collected from 1990 to 2001 were group 1 and those from 2002 to 2011 were group 2. The patients' characteristics of the two groups and clinical findings such as demography, bacteriology, and underlying comorbid diseases were reviewed to analyze the major changes in etiology and epidemiology between two decades. The goal of this study was to analyze whether difference existed between the two groups, especially with regard to the diagnostic criteria of Cohen and Friedman. SPSS version 17.0 was used for data management and statistical analysis. Univariate analyses were performed using Student's* t*-test (for continuous variables) or the chi-square or Fisher's exact tests (for categorical variables); *P* < 0.05 was considered statistically significant. This study was undertaken with the approval of the Research Ethics Committee of the Taipei Veteran General Hospital.

## 3. Results

During the study period, a total of 55 patients with the diagnosis of osteomyelitis of the temporal bone were treated at our institution. Group 1 included 20 patients with the remaining 35 patients assigned to group 2. Group 2 was further divided into group 2-only patients without previous ear operation and group 2-only patients with previous ear operation. Among group 1, there were 13-male and 7-female patients, with a mean age of 64.3 ± 9.9 years. As for group 2, there were 21 males and 14 females, with a mean age of 66.8 ± 15.0 years. There was no difference between these groups with respect to gender and age, but between-group difference was noted between group 1 and group 2-only patients without previous ear operation (64.3 ± 9.9 years versus 71.8 ± 12.7 years; *P* = 0.044) and between group 2-only patients with previous ear operation and group 2-only patients without previous ear operation (60.1 ± 15.7 years versus 71.8 ± 12.7 years; *P* = 0.021).

There were five patients with previous radiotherapy (four patients with nasopharyngeal carcinoma and one patient with basal cell carcinoma at external auditory canal). Three patients were in group 2-only without previous ear operation and another two in group 2-only patients with previous operation. All five patients had no history of diabetes mellitus. As compared with group 1, patients with previous radiotherapy had lower prevalence of diabetes mellitus (95% versus 0%; *P* < 0.001), and fewer presented with granulation at EAC and otitis externa (80% versus 20%, *P* = 0.023; 100% versus 20%, *P* < 0.001).

Table 1 shows the comparison among these groups with osteomyelitis of the temporal bone. The prevalence of diabetes was higher in group 1 than in group 2 (95.0% versus 45.7%; *P* < 0.001), group 2-only patients without previous ear operation (95.0% versus 60.0%; *P* = 0.020), and group 2-only patients with previous ear operation (95.0% versus 26.7%; *P* < 0.001). All patients except one in group 1 presented with otalgia, and all patients in group 1 had swelling external ear canal (EAC). In contrast to group 1, otalgia was noted only in 22 and 3 patients of group 2 and group 2-only patients with previous ear operation, respectively. Significant differences were noted between group 1 versus group 2 and group 1 versus group 2-only patients with previous ear operation, respectively (95% versus 62.9%, *P* = 0.010, and 95% versus 20.0%, *P* < 0.001). Interestingly, between-group difference was noted between group 2-only patients without previous operation and group 2-only patients with previous operation (*P* < 0.001). However, between-group difference was not noted between group 1 and group 2-only patients without previous ear operation. As for otitis externa, fewer patients who presented with otitis externa were noted in group 2, group 2-only patients without previous ear operation, and group 2-only patients with previous ear operation (*P* < 0.001, *P* = 0.02, and *P* < 0.001, resp.). EAC granulation was more common in group 1 patients (80% in group 1 versus 31.4% in group 2; *P* = 0.001). Significant differences were also renowned in group 2-only patients without previous operation and group 2-only patients with previous operation (*P* = 0.048 and *P* < 0.001, resp.). EAC granulation was also less common in group 2-only patients with previous operation, compared with group 2-only patients without previous operation (*P* = 0.002). However, there was no between-group difference in discharge over EAC (90% and 85.7% in groups 1 and 2, resp.; *P* = 1.000).

Patients in group 1 were also characterized by significantly higher rates of positive culture for* Pseudomonas aeruginosa* (55% versus 25.7%; *P* = 0.043). Significantly lower rates of positive culture for* Pseudomonas aeruginosa* were also noted in group 2-only patients with previous operation (55% versus 0%; *P* = 0.001) but not in group 2-only patients without previous operation. Although increased methicillin-resistant* Staphylococcus aureus* (MRSA) infection was noted in group 2 patients, there was no between-group difference in the number of MRSA infections (25% and 40.0% in groups 1 and 2, resp.; *P* = 0.378). Overall, the most common pathogen was* Pseudomonas aeruginosa* and MRSA in both groups.

Laboratory data were also collected. Leukocytosis was only noted in 0% to 11.1% patients of all groups. 50% to 80% patients with elevated ESR and 66.7% to 78.6% patients with increased CRP were noted among these groups. But there was no between-group difference that was renowned between these groups.

In Table 2, we classified these patients into three groups: patients with* P. aeruginosa *infection, non-*P. aeruginosa *infection-only patients without previous ear operations, and non-*P. aeruginosa* infection-only patients with previous ear operations. Parameters clearly differentiating patients infected with* Pseudomonas aeruginosa* from nonpseudomonas infection were associated with higher prevalence of diabetes (80.0% in patients with pseudomonas infection versus 54.3% in nonpseudomonas infection; *P* = 0.082), and patients with pseudomonas infection also more commonly complained of otalgia, otorrhea, and otitis externa (otalgia 100% versus 60.0%, *P* = 0.001; otorrhea 100% versus 80.0%, *P* = 0.040; and otitis externa 85% versus 54.3%, *P* = 0.038). Moreover, all patients infected with* Pseudomonas aeruginosa* had no previous ear operation (*P* < 0.001). However, there was no between-group difference after removing patients with previous operation from nonpseudomonas infection group, except otorrhea (*P* = 0.02). Furthermore, non-*P. aeruginosa*-only patients with previous ear operation have lower prevalence of diabetes (*P* = 0.002) and less complained of otalgia, otitis externa, and granulation at EAC (*P* < 0.001, *P* < 0.001, and *P* = 0.005, resp.). Among non-*P. aeruginosa* infection patients, patients with previous ear operations were also characterized with lower prevalence of diabetes (*P* = 0.007) and less complained of otalgia, otitis externa, and granulation at EAC (*P* < 0.001, *P* < 0.001, and *P* = 0.007, resp.).

Of a total of 55 patients with temporal bone osteomyelitis, 15 patients had previous ear operation (2 ventilation tube insertions, 1 CO_2_ laser operation, 1 type I tympanoplasty, and 11 tympanomastoidectomies). As shown in Table 3, these patients were characterized with lower prevalence of diabetes (26.7% versus 77.5%; *P* = 0.001) and younger age (60.1 ± 15.7 years versus 68.0 ± 11.9 years; *P* = 0.05) and they also less frequently suffered from otalgia, otitis externa, and granulation in EAC (20% versus 95.0%, *P* < 0.001; 13.3% versus 85.0%, *P* < 0.001; and 13.3% versus 62.5%, *P* = 0.002). As for microbiology results,* Pseudomonas aeruginosa* was not identified (0%) and MRSA was identified in seven (46.7%), nontuberculous mycobacteria (NTM) in three (20.0%), negative culture in two (13.3%), and others in three (20.0%).

## 4. Discussion

In the past, osteomyelitis of the temporal bone was seen as mostly caused by and secondary to MOE that was characterized by severe swelling in EAC and granulation tissue in the floor of the ear canal at the bony-cartilaginous junction [2, 8, 11]. Hence, the diagnosis of osteomyelitis of the temporal bone frequently followed the diagnostic criteria of MOE proposed by Cohen and Friedman [8]. Among the criteria, pain, exudate, edema, and granulation tissue of EAC and positive Tc99m scan were obligatory signs that occurred in all patients of MOE. Frequently, MOE was considered as the equivalent of osteomyelitis of the temporal bone.

**Figure 1:**
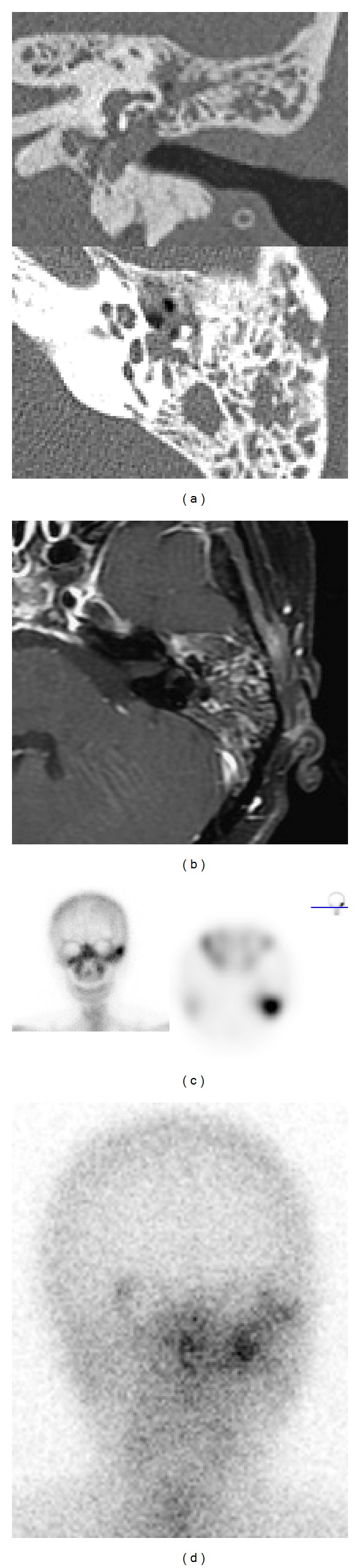
Images of a patient of temporal bone osteomyelitis after radiotherapy for nasopharyngeal carcinoma. (a) CT. (b) Contrast-enhanced T1-weighted MR. (c) Tc99m bone scan. (d) Gallium scan. See text for details.

However, these reliable clinical signs of osteomyelitis of the temporal bone have been altered in the current period with the absence of granulation of EAC in some cases [9] and painless remainder [10]. As shown in our study, appearance of otitis externa and granulation over EAC decreased in the past 10 years (Table 1, *P* < 0.001 and *P* = 0.001, resp.). This might indicate that, at least in these patients, osteomyelitis was not secondary to infection of the EAC. For example, a 53-year-old nondiabetic female patient with history of nasopharyngeal carcinoma received radiotherapy 5 years before onset of ear symptoms. Insertion of ventilation tube was performed due to recurrent otitis media with effusion. Purulent left ear discharge and otalgia were noted for 1 year. Axial CT image demonstrated increased soft tissue in left tympanic cavity and mastoid area with mild bony destruction (Figure 1(a)). Contrast-enhanced axial T1-weighted MRI revealed inflammatory process over left mastoid area (Figure 1(b)). Bone scan (Tc99m) revealed increased uptake at left temporal bone area (Figure 1(c)). Gallium scan demonstrated intense gallium uptake in left temporal bone (Figure 1(d)). No infection or swelling of EAC was noted. The traditional theory of infection spread through bony fissures from EAC to temporal bone no longer applies in these patients. This difference still existed in group 2-only patients without previous ear operation (Table 1, *P* = 0.020 and *P* = 0.048, resp.) and group 2-only patients with previous ear operation (Table 1, *P* < 0.001 and *P* < 0.001, resp.). Additionally, patients with previous radiotherapy also less presented with granulation at EAC and otitis externa. In fact, this result means that osteomyelitis of the temporal bone has been changing and was not always a disease secondary to MOE and direct extension of infection from bony parts of the middle ear and mastoid to deep temporal bone structures provides a possible explanation. These significant differences of clinical features may be due to previous ear operations, previous radiotherapy, and influence of the time.

**Figure 2:**
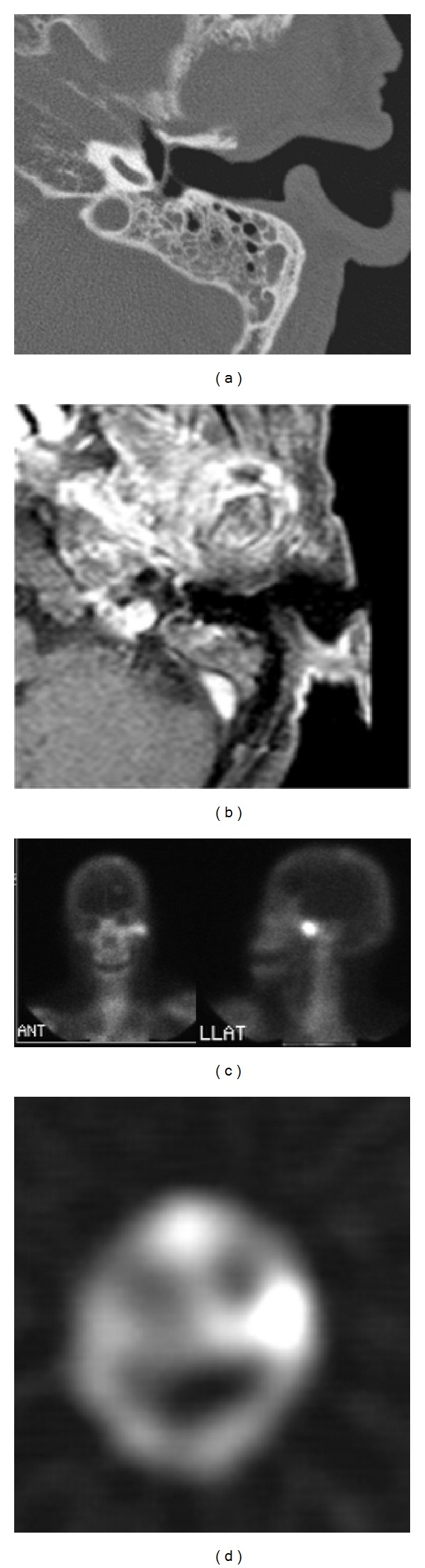
Images of a patient of temporal bone osteomyelitis without history of DM. (a) CT. (b) Contrast-enhanced T1-weighted MR. (c) Tc99m bone scan. (d) Gallium scan. See text for details.

The epidemiology of osteomyelitis of the temporal bone has also changed in the past 10 years. Osteomyelitis of the temporal bone was predominantly seen in elderly and diabetes mellitus in the past. It has been postulated that microangiopathy in the ear canal predisposes elderly diabetic patients to MOE [5]. Previous review has quoted the prevalence of diabetes in MOE cases as 86–90% [5] and analogous to group 1 patients in our study. In contrast to previous study, decreased incidence of diabetes in group 2 patients was well known in the present study (Table 1; 45.7% in group 2; *P* < 0.001) and even after excluding patients with previous ear surgery (Table 1; 60% in group 2-only patients without previous ear operation; *P* = 0.020). Old age (>55) is an occasional criterion of Cohen and Friedman. The mean age was even older in our study (Table 1; 64.3 ± 9.9 years in group 1 versus 71.8 ± 12.3 years in group 2-only patients without previous ear operation; *P* = 0.044). Therefore, we must keep in mind that osteomyelitis of the temporal bone should be considered in nondiabetic and older patients. For instance, a 73-year-old male patient without history of DM or immunocompromised status suffered from otalgia of left ear for 2 weeks. Axial CT image showed bony destruction of left temporomandibular (TM) joint, but no otitis externa (Figure 2(a)). Contrast-enhanced axial T1-weighted MRI revealed inflammatory process over left TM joint and mastoid area (Figure 2(b)). Bone scan (Tc99m) revealed increased uptake at left temporal bone area (Figure 2(c)). Gallium scans with single photon emission computed tomography (SPECT) demonstrated intense gallium uptake in left temporal bone (Figure 2(d)).

**Figure 3:**
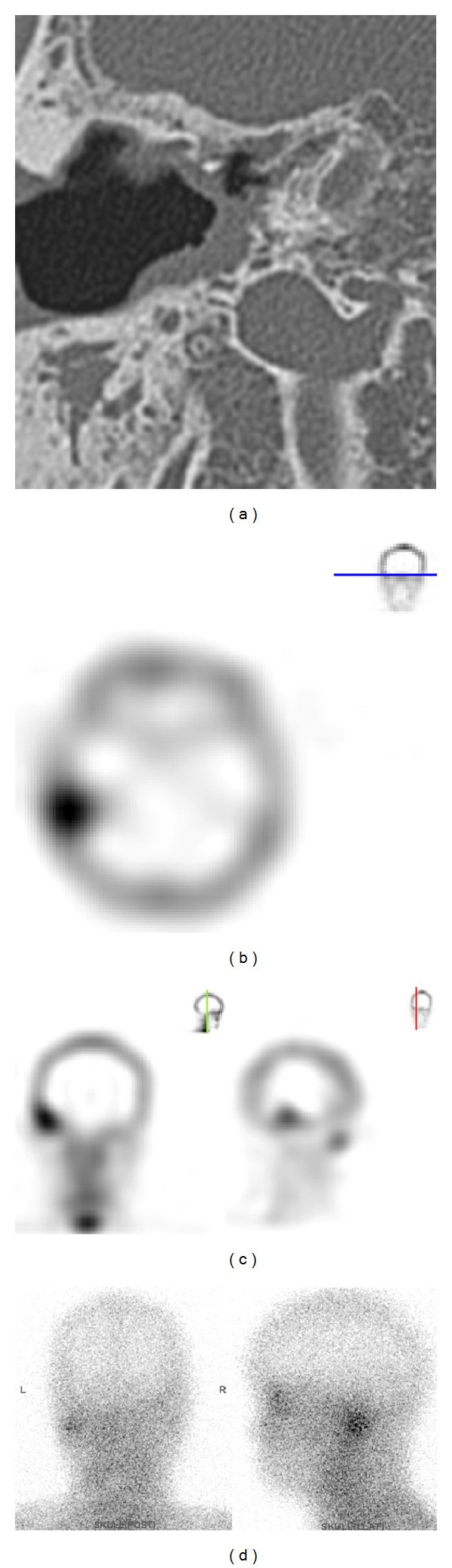
Images of a patient of temporal bone osteomyelitis after surgery for chronic otitis media with cholesteatoma. (a) CT. (b, c) Tc99m bone scan. (d) Gallium scan. See text for details.

In this series, patients with previous ear operation were characterized by lower prevalence of diabetes (Table 3, *P* = 0.001), younger age (60.1 ± 15.7 years versus 68.0 ± 11.9 years; *P* = 0.05), and fewer patients complained of otalgia (*P* < 0.001). All of these findings were completely different from the classic diagnostic criteria. These patients most commonly presented with painless otorrhea. As for local findings, otitis externa and granulation in EAC were not common in patients with previous operation (*P* < 0.001 and *P* = 0.002, resp.). Surprisingly, the most common pathogen was MRSA (46.7%), followed by NTM, and* Pseudomonas aeruginosa* was not isolated in this group. Based on the bacteriology of our study, whether systemic antipseudomonal antibiotics still remain the primary therapy for this group of patients is questionable. Antibiotics that are effective against MRSA may be considered alternatively. A possible explanation is that otologic surgery may change the anatomic structures of external auditory canal and middle ear cavity, consequently resulting in different infectious sources, pathways, pathogens, and clinical manifestations as described in our study, and we should pay more attention to these changes. However these patients were characteristically of younger age and low prevalence of diabetes and presented with painless otorrhea, which was absolutely different from classic clinical presentations. As shown here, a 28-year-old nondiabetic female patient received atticomastoidectomy and modified type III tympanoplasty for chronic otitis media with cholesteatoma. Painless ear discharge developed and persisted for several months after surgery. Axial CT image showed soft tissue lesion at right mastoid open cavity (Figure 3(a)). Tc99m scan revealed increased uptake over right temporal bone (Figures 3(b) and 3(c)). Gallium scan demonstrated uptake over right temporal area (Figure 3(d)). Bacterial culture grew MRSA.

**Figure 4:**
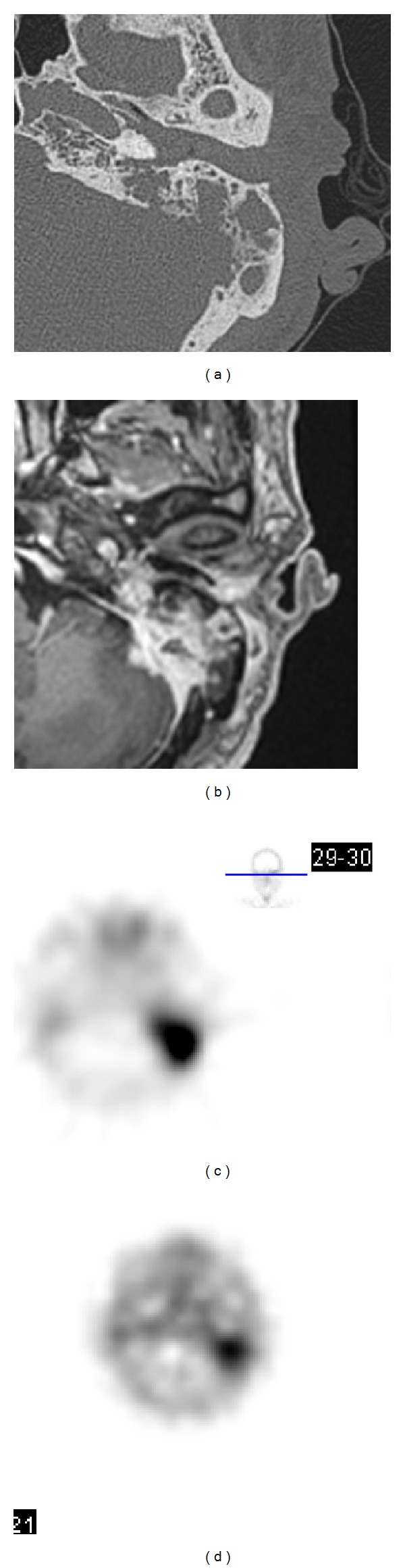
Images of a patient of temporal bone osteomyelitis caused by nontuberculous mycobacteril infection. (a) CT. (b) Contrast-enhanced T1-weighted MR. (c) Tc99m bone scan. (d) Gallium scan. See text for details.

*Pseudomonas aeruginosa* was reported to be the causal organism in nearly all cases of MOE [8]. In diabetic patients, poor vascular supply resulting from microvascular disease is aggravated by pseudomonal vasculitis, which further restricts tissue perfusion [12]. This higher prevalence of diabetes mellitus was observed in pseudomonas infection patients although there was no between-group difference (Table 2, 80% versus 54.3%; *P* = 0.082). In contrast, osteomyelitis of the temporal bone caused by pseudomonas infection was much less frequently found in group 2 patients (Table 1, *P* = 0.043). Lower prevalence of diabetes and higher incidence of previous otologic surgery in group 2 patients might explain this difference between groups. Our findings highlight the fact that osteomyelitis of the temporal bone is not an infection caused by* Pseudomonas aeruginosa* alone; other infectious organisms such as MRSA and NTM also play a more and more important role in the current period. For instance, Figure 4 shows an 83-year-old male patient of temporal bone osteomyelitis that was caused by NTM infection. Axial CT image demonstrates soft tissue density lesion over left external auditory canal with bony destruction of the left temporal bone (Figure 4(a)). Contrast-enhanced axial T1-weighted MRI showed local meningitis and cerebellitis (Figure 4(b)). Bone scan (Tc99m) revealed increased uptake at left temporal bone area (Figure 4(c)). Gallium scans with SPECT demonstrated intense gallium uptake in left temporal bone (Figure 4(d)).

In this series, leukocytosis was not a good diagnostic tool because only 0%–11.1% of patients of all these groups had leukocytosis. Elevated ESR value and increased CRP level were noted at 50% to 80% and 66.7% to 78.6% patients of these groups, respectively. As a result, inflammation makers (ESR and CRP) may be a useful laboratory test for temporal bone osteomyelitis. An elevated erythrocyte sedimentation rate (ESR) was identified as a useful tool in screening for this illness and monitoring response to therapy [5, 13].

Although severe otalgia has long been regarded as a hallmark of MOE, only 62.9% of patients in group 2 presented with otalgia and between-group difference was observed (Table 1, *P* = 0.010). This difference became insignificant after excluding patients with previous surgery from group 2 (*P* = 1.000). Additionally, significant difference was noted between group 2-only patients without previous ear operation and group 2-only patients with previous ear operations (Table 1; 95.0% versus 20.0%; *P* < 0.001). Obviously, this discrepancy came from painless osteomyelitis of the temporal bone in some patients after surgery. Thus, clinical findings in patients with previous ear operation were completely different from those without previous ear operation (Tables 1 and 3). As mentioned above, the previous diagnostic criteria did not quite agree with osteomyelitis of the temporal bone due to the changing traits. Hence, early detection of patients with atypical symptoms and signs has become a diagnostic challenge. Among the various image studies, bone scan, including Tc99m scan and gallium scan, is considered as a useful tool for initial diagnosis of osteomyelitis of the temporal bone. Tc99m scan is exquisitely sensitive because the radiotracer accumulates at sites of osteoblastic activity but is relatively nonspecific [14] and also remains positive until cessation of osteoblastic activity that persists long after the infection has been eradicated. Thus, it had limited role when assessing the status of acute osteomyelitis and its response to therapy [15]. In contrast, gallium citrate is absorbed by macrophages and reticular endothelial cells and concentrates in areas of active inflammation, including soft tissue and bone infections. Gallium scans quickly return to normal after the infection has settled and can be used for radiological assessment of the response to therapy [7]. Gallium scan is more specific to acute infection, and its SPECT technique can be used in the early diagnosis, follow-up evaluation, and prognosis for osteomyelitis of the temporal bone [16, 17]. In clinical practice, patients with positive Tc99m and negative gallium results usually turned out to have limited mastoiditis or postoperative changes such as mastoidectomy cavity infection, not osteomyelitis of the temporal bone. On the other hand, the clinical courses of patients with positive gallium scans and Tc99m scans generally indicate inflammation of the temporal bone. Accordingly, our results suggest that both gallium and Tc99m scans should be performed earlier in patients with high suspicion because the clinical manifestations of osteomyelitis of the temporal bone have become less typical and noticeable than before.

## 5. Conclusion

In this paper, we have demonstrated the changing traits of osteomyelitis of the temporal bone and brought up several findings. Osteomyelitis of the temporal bone was not always related to MOE. The diagnostic criteria of MOE did not comfort to atypical patients. Clinicians must establish earlier diagnosis and treatment for osteomyelitis of the temporal bone, even in nondiabetic and immunocompetent patients, and avoid more severe complications. It is worthy of mention that patients with previous otologic surgeries had pictures that were thoroughly different from typical diagnostic criteria and mostly presented with painless otorrhea and had different microbiology.

## Figures and Tables

**Table 1:** Comparison among group 1, group 2, group 2-only patients without previous operation (group 2 without op), and group 2-only patients with previous operation (group 2 with op).

	Group 1 (*n* = 20)	Group 2 (*n* = 35)	Group 2 without op (*n* = 20)	Group 2 with op (*n* = 15)	Significance			
					Group 1 versus group 2	Group 1 versus group 2 without op	Group 1 versus group 2 with op	Group 2 without op versus group 2 with op
Clinical features								
Age (mean ± SD), years	64.3 ± 9.9	66.8 ± 15.0	71.8 ± 12.7	60.1 ± 15.7	0.454	**0.044**	0.351	**0.021**
Gender (M/F)	13/7	21/14	15/5	6/9	0.779	0.731	0.182	0.080
Diabetes	19 (95.0%)	16 (45.7%)	12 (60.0%)	4 (26.7%)	**<0.001**	**0.020**	**<0.001**	0.087
Previous surgery	0 (0%)	15 (42.9%)	0 (0%)	15 (100%)	**<0.001**	—	—	—
Otalgia (pain)	19 (95.0%)	22 (62.9%)	19 (95.0%)	3 (20%)	**0.010**	1.000	**<0.001**	**<0.001**
Otorrhea (exudate)	18 (90%)	30 (85.7%)	16 (80.0%)	14 (93.3%)	1.000	0.661	1.000	0.365
Otitis externa (swelling)	20 (100%)	16 (45.7%)	14 (70.0%)	2 (13.3%)	**<0.001**	**0.020**	**<0.001**	0.069
Granulation over EAC	16 (80%)	11 (31.4%)	9 (45.0%)	2 (13.3%)	**0.001**	**0.048**	**<0.001**	**0.002**
CN involvement	4 (20%)	5 (14.3%)	5 (25.0%)	1 (6.7%)	0.709	1.000	0.680	0.672
Microbiology								
*P. aeruginosa *	11 (55%)	9 (25.7%)	9 (45.0%)	0 (0%)	**0.043**	0.752	**0.001**	**0.004**
MRSA	5 (25%)	14 (40.0%)	7 (35.0%)	7 (46.7%)	0.378	0.731	0.282	0.511
Negative culture	2 (10%)	3 (8.6%)	1 (5.0%)	2 (13.3%)	1.000	1.000	1.000	0.565

Bold type denotes statistically significant difference (*P* < 0.05).

**Table 2:** Comparison among patients with *P. aeruginosa* infection, non-*P. aeruginosa* infection, non-*P. aeruginosa* infection-only patients without previous ear operation (non-*P. aeruginosa* infection without op), and non-*P. aeruginosa* infection-only patients with previous ear operation (non-*P. aeruginosa* infection with op).

	*P. aeruginosa * (*n* = 20)	Non-*P. aeruginosa * (*n* = 35)	Non-*P. aeruginosa *without op (*n* = 20)	Non-*P. aeruginosa* with op (*n* = 15)	Significance			
					*P. aeruginosa* versus non-*P. aeruginosa *	*P. aeruginosa* versus non-*P. aeruginosa* without op	*P. aeruginosa* versus non-*P. aeruginosa* with op	Non-*P. aeruginosa* without op versus non-*P. aeruginosa* with op
Clinical features								
Age (mean ± SD), years	65.8 ± 13.1	65.9 ± 13.7	70.3 ± 10.5	60.1 ± 15.7	0.976	0.242	0.252	**0.029**
Gender (M/F)	13/7	21/14	15/5	6/9	0.779	0.731	0.182	0.080
Diabetes	16 (80%)	19 (54.3%)	15 (75%)	4 (26.7%)	0.082	1.000	**0.002**	**0.007**
Previous surgery	0 (0%)	15 (42.9%)	0 (0%)	15 (100%)	**<0.001**	—	—	—
Otalgia (pain)	20 (100%)	21 (60.0%)	18 (90%)	3 (20%)	**0.001**	0.487	**<0.001**	**<0.001**
Otorrhea (exudate)	20 (100%)	28 (80.0%)	14 (70%)	14 (93.3%)	**0.04**	**0.020**	0.429	0.199
Otitis externa (swelling)	17 (85%)	19 (54.3%)	17 (85%)	2 (13.3%)	**0.038**	1.000	**<0.001**	**<0.001**
Granulation over EAC	13 (65%)	14 (40.0%)	12 (60%)	2 (13.3%)	0.097	1.000	**0.005**	**0.007**
CN involvement	3 (15%)	5 (14.3%)	4 (20%)	1 (6.7%)	1.000	1.000	0.619	0.365

Bold type denotes statistically significant difference (*P* < 0.05).

**Table 3:** Patient characteristics between patients with and without history of previous otologic surgery.

	Previous surgery (*n* = 15)	No previous surgery (*n* = 40)	Significance
Clinical features			
Age (mean ± SD), years	60.1 ± 15.7	68.0 ± 11.9	*P* = 0.05
Gender (M/F)	6/9	28/12	*P* = 0.062
Diabetes	4 (26.7%)	31 (77.5%)	*P* = 0.001
Otalgia (pain)	3 (20%)	38 (95.0%)	*P* < 0.001
Otorrhea (exudate)	14 (93.3%)	34 (85.0%)	NS
Otitis externa (swelling)	2 (13.3%)	34 (85.0%)	*P* < 0.001
Granulation over EAC	2 (13.3%)	25 (62.5%)	*P* = 0.002
CN involvement	1 (6.7%)	7 (17.5%)	NS
Microbiology			
*Pseudomonas aeruginosa *	0 (0%)	20 (50.0%)	*P* < 0.001
MRSA	7 (46.7%)	12 (30.0%)	NS
NTM	3 (20%)	1 (2.5%)	*P* = 0.057
Negative	2 (13.3%)	3 (7.5%)	NS
